# Cenobamate, a Sodium Channel Inhibitor and Positive Allosteric Modulator of GABA_A_ Ion Channels, for Partial Onset Seizures in Adults: A Comprehensive Review and Clinical Implications

**DOI:** 10.3390/neurolint13020026

**Published:** 2021-06-09

**Authors:** Dustin R. Latimer, Amber N. Edinoff, Rachel D. Ruff, Kelsey C. Rooney, Kayla M. Penny, Shaan B. Patel, Suresh Sabbenahalli, Adam M. Kaye, Elyse M. Cornett, Omar Viswanath, Ivan Urits, Alan D. Kaye

**Affiliations:** 1 Department of Psychiatry and Behavioral Medicine Louisiana State University Health Science Center Baton Rouge, Baton Rouge, LA 70808, USA; dlatim@lsuhsc.edu (D.R.L.); ssabb1@lsuhsc.edu (S.S.); 2 Department of Psychiatry and Behavioral Medicine, Louisiana State University Health Science Center Shreveport, Shreveport, LA 71103, USA; 3 School of Medicine, Louisiana State University Health Sheveport, Sheveport, LA 71103, USA; rruff@lsuhsc.edu (R.D.R.); kroone@lsuhsc.edu (K.C.R.); kpenny@lsuhsc.edu (K.M.P.); spat25@lsuhsc.edu (S.B.P.); 4 Thomas J. Long School of Pharmacy and Health Sciences, Department of Pharmacy Practice, University of the Pacific, Stockton, CA 94103, USA; akaye@PACIFIC.EDU; 5 Department of Anesthesiology, Louisiana State University Health Shreveport, Shreveport, LA 71103, USA; ecorne@lsuhsc.edu (E.M.C.); ivanurits@gmail.com (I.U.); akaye@lsuhsc.edu (A.D.K.); 6 College of Medicine-Phoenix, University of Arizona, Phoenix, AZ 85004, USA; viswanoy@gmail.com; 7 Department of Anesthesiology, Creighton University School of Medicine, Omaha, NE 68124, USA; 8 Valley Anesthesiology and Pain Consultants, Envision Physician Services, Phoenix, AZ 85004, USA; 9 Southcoast Physicians Group Pain Medicine, Southcoast Health, Wareham, MA 02720, USA

**Keywords:** partial seizures, epilepsy, Cenobamate, antiepileptic drug, sulfamoylphenyl moieties, XCOPRI, GABA_A_ ion channels

## Abstract

Medical management of epilepsy seeks to eliminate or to reduce the frequency of seizures, help patients maintain a normal lifestyle, and maintain psychosocial and occupational activities, while avoiding the negative side effects of long-term treatment. Current FDA approved drugs have been shown to have similar efficacy; however, they all share a commonality of having side effects that have the potential to significantly reduce a patient’s quality of life. Cenobamate, a newly-FDA approved drug used to treat partial-onset seizures in adult patients, has demonstrated promise in that it works on two proposed mechanisms that are commonly associated with epilepsy. Cenobamate acts as a positive allosteric modulator of the GABA_A_ ion channels and is effective in reducing repetitive neuronal firing by inhibition of voltage-gated sodium channels, although the complete mechanism of action is currently unknown. The efficacy of Cenobamate with its low toxicity and adverse drug reaction profile emphasizes the need to further evaluate antiepileptic therapies containing sulfamoylphenyl and/or carbamate moieties in their chemical structure. Recent studies have found more patients to be seizure free during the maintenance period when compared to placebo. The most common side effects reported in with Cenobamate are somnolence, dizziness, headache, nausea, and fatigue. There are currently ongoing phase III studies looking to further evaluate the long-term benefits of Cenobamate and investigate adverse events.

## 1. Introduction

Seizure is often classified and further subclassified to describe paroxysmal events of sudden onset neuronal firing resulting in a short period of altered neurologic function. There are many causes for seizures and seizure mimicking events such as syncope, migraine, stroke, and psychogenic nonepileptic seizures. After ruling out seizure mimickers, the management of a new-onset, single seizure is driven by the determination of the underlying cause. Seizures can result from an external cause (e.g., nonepileptic) or an intrinsic dysfunction of the central nervous system. A baseline metabolic evaluation should be done to evaluate the cause of a new seizure. This workup should start with an evaluation of a patient’s complete metabolic panel and evaluation of medications that can cause a disruption of metabolites causing a seizure or a medication that is known to have seizures as a potential adverse reaction. An initial evaluation should also determine the likelihood that a patient will have additional seizures, assist in the decision whether to begin antiseizure drug therapy, and direct appropriate treatment to the underlying cause, if identified. Once any treatable, systemic process is ruled out, as well as any treatable underlying brain pathology, the identification of factors that increase the likelihood of recurring seizures is often made. Then, a diagnosis of epilepsy may be considered.

Epilepsy is often diagnosed after an individual has two or more unprovoked seizures greater than 24 h apart and can be explained as a disorder of an enduring predisposition to generalized epileptic seizures [1]. In addition to this criteria, two other conditions that are used clinically to diagnose epilepsy are a diagnosis of an epilepsy syndrome or one unprovoked seizure and a probability of further seizures similar to the general recurrence risk, at least 60%, after two unprovoked seizures, occurring over the next ten years [1].

The seizures occurring in epilepsy are often further classified into focal or generalized. Generalized seizures refer to sudden onset neuronal firing affecting millions of neurons in both hemispheres of the brain, typically with a loss of consciousness. A focal seizure is defined by the sudden onset of neuronal firing with a very specific region of the brain that may or may not result in the loss of consciousness. The degree of consciousness affected in a focal seizure is often categorized as a focal seizure with awareness, impaired awareness, or unknown awareness. Focal seizures can also be described as having a motor or nonmotor component of the event. In a motor seizure, the predominating symptoms involve motor activity, with sustained muscle tone, jerking, or atonic periods. Non-motor seizures are often referred to as absence seizures due to the characteristic manifestation of staring into space. Though generalized seizures are defined as having a loss of consciousness, they can also be broken down into motor and nonmotor seizures.

While there has been a long-standing classification of epilepsies as focal or generalized, two additional categories were added in 2017: generalized and focal epilepsy and unknown if generalized or focal epilepsy [1]. These additions allow for the overlap that often occurs between focal and generalized seizures, as many focal seizures (including awareness and impaired awareness subtypes) may start focally but spread diffusely and become a generalized seizure. The primary goal of this extensive classification system is to aid in the research and development of antiepileptic treatments while maintaining a digestible structure of the organization to epilepsy [2].

Treatment of epilepsy with antiseizure medication is frequently reserved for individuals who meet the criteria for a diagnosis of epilepsy, but in patients with a single unprovoked seizure, it is difficult to assess the risk of recurrent seizures [2]. The initiation of treatment is often subjected to a clinician’s judgment if the patient does not clearly meet the criteria for a diagnosis of epilepsy [3]. Adults presenting with an unprovoked first seizure and who immediately begin antiseizure medication reduces the risk of seizure recurrence by approximately 35 percent over the next one to two years and almost half of these patients on their first antiseizure drug trial will become seizure-free [2]. Finding an antiseizure medication that successfully balances efficacy and adverse effects has proven to be a difficult task. There are approximately thirty antiepileptic medications to choose from, and no single medication has been proven to be marginally more effective with minimal adverse reactions than other antiepileptic medications [2]. Current FDA approved drugs have been shown to have similar efficacy, but they all also share a commonality of having side effects that have the potential to significantly reduce a patient’s quality of life.

## 2. Epidemiology of New-Onset Adult Epilepsy

Seizures affect eight to ten percent of the general population in a lifetime [4,5]. One to two percent of emergency room visits are for seizures and of those seizures, 26% are new-onset [6]. Almost half of new-onset seizures will present in adults over 65 years of age [7,8]. There is a strong association between age and risk for developing epilepsy [9]. The incidence of new-onset epilepsy in adults over age 65, in various studies, is one to three out of every 1000 people per year; this is two to six times higher than in the younger adult populations [8,9,10,11,12,13]. Epilepsy prevalence is two to five percent in adults over age 65, which is three to four times higher in younger adult populations [11,12]. Developing epilepsy does not seem to differ greatly between genders. The incidence of adult, new-onset epilepsy is slightly higher in females than in males [7,11].

A 2001 to 2005 study of US Medicare beneficiaries over age 65 demonstrated average annual incident rates were stratified by race. African Americans had the highest at 4.1 per 1000, then whites at 2.3 per 1000, and Asians and Native Americans were the lowest at 1.6 and 1.1 per 1000, respectively [11]. A similar trend between races in younger and older populations was shown in other studies, but the association between them remains unexplained [12].

## 3. Pathophysiology of Epilepsy

Seizures are a hyperexcitable and hypersynchronous manifestation of a pathological imbalance between excitatory and inhibitory factors (E/I imbalance) in neurotransmission [14]. Epilepsy is defined as recurrent, unprovoked seizures. Consideration of the E/I imbalance can help guide an understanding of the pathophysiological mechanisms of epilepsy. In adults, this balance is primarily struck between glutamate (an excitatory neurotransmitter), GABA (an inhibitory neurotransmitter), and the extracellular potassium concentration [14]. Phenobarbital and benzodiazapenes have historically been used to prevent seizures or end an actively seizing patient’s seizure by working on the GABA transmission mechanism [15]. Structural and physiologic abnormalities that alter the level of these mediators influence the development of epileptic seizures. These abnormalities can be genetic or acquired [16]. Mechanisms of these aberrancies can be attributable to an array of neurovascular accidents such as traumatic, anoxic, inflammatory, genetic, or cryptogenic causes. Cryptogenic etiologies account for 25 to 50% of epilepsy cases [4,10,13,17]. Neurovascular etiologies of seizures account for approximately 50% of new-onset epilepsy in adults over 65 [13]. These conditions create structural and functional changes that lead to epilepsy.

Alterations in the E/I balance can occur at any level ranging from genetic mutations to structural abnormalities in neuronal circuits. Genetic and structural pathologies leading to epilepsy include abnormal ion channel function, abnormal neurotransmitter transport proteins, abnormal neurotransmitter receptor function, and abnormal synaptic connectivity [14,16]. Abnormal inward rectifying potassium channels, primarily within astrocytes, lead to a high level of extracellular potassium after neuronal firing. Increases in extracellular potassium result in neuronal hyperexcitability [14]. Glial cells have membrane transporters responsible for removing extracellular glutamate to maintain healthy brain function; dysfunction in these proteins can cause hyperexcitability resulting in epileptic seizures [14].

Dysregulation of GABAergic transmission is theorized to play a role in epileptogenesis [14]. A well-known example of this is seen in Angelmann syndrome, where there are documented abnormalities within GABA receptor subunits associated with epilepsy [16]. Inhibition of GABA transmission is associated with paroxysmal depolarization shift (PDS); barbiturates and benzodiazapenes utilize this as their mechanism of action [15]. PDS is characterized by abnormal fluctuations in neuronal membrane potential seen after an initial depolarization by synaptic forces [14,18]. Two emerging ideas behind PDS are the “synaptic theory” and the “epileptic-neuron theory” [18]. The synaptic theory states excessive stimulation of previously normal-functioning neurons via an aberrant recurrent synaptic feedback which causes abnormal depolarizations. The epileptic-neuron theory asserts that the intrinsic neuronal properties of affected neurons alter conductance resulting in PDS [18]. The termination of a PDS is mediated by potassium and chloride conductance and GABA receptors. Failure to terminate a PDS will result in a seizure [14]. Ultimately, seizures are manifestations of a complex constellation of disturbances at multiple levels of cell structure and function and of large neural network activity [14]

## 4. Risk Factors, Diagnosis, and Presentation of Epilepsy

Initial diagnostic evaluation for a first seizure should start with a complete history and physical examination (H&P). The goal of the complete H&P is to characterize the presentation of the seizure event, rule out other diagnoses, discern whether prior seizures have occurred, and assess for underlying risk factors in the family history, past medical history, and medications [19].

A history usually delineates between a focal and generalized seizure based on information of preictal events, ictal behaviors, and postictal state. Asking directed questions about circumstances just prior to a seizure can identify potential seizure precipitants or triggers such as strong emotions, loud music, intense exercise, and flashing lights [20,21]. The presentation of most seizures is abrupt onset, usually within seconds, and resolves within a couple of minutes, for both generalized and focal seizures. Seizures that do not terminate within a couple of minutes can indicate alternative conditions that are nonepileptic or a potentially fatal outcome in an individual experiencing status epilepticus [19]. Alteration of consciousness is associated with a generalized seizure; focal seizures can vary in their disruption of consciousness. Patients with focal seizures without alteration in consciousness can describe the seizure while some individuals experiencing a focal seizure may have an impaired sense of consciousness and cannot recall the event. Focal seizures typically present with localizing behaviors. For example, dysphasia insinuates the involvement of the dominant hemisphere. The postictal period should last between 10 and 20 min and gradually resolve. A prolonged postictal state could indicate residual seizure activity [19].

Physical examination is usually useful for identifying the causes of provoked seizures. One of the most specific indicators of a seizure, more typically a tonic-clonic seizure, is lateral tongue biting [22]. Risk factors for epilepsy include family history and previous epileptic seizures. Past medical history can elucidate identifiable triggers, and events such as trauma, specifically head trauma, may manifest as epilepsy later in life.

Laboratory studies include basic electrolytes, calcium, magnesium, glucose, complete blood count, renal function, liver function, urinalysis, and toxicology screens to rule out physiologic causes. Abnormal sodium is one of the more common electrolyte disturbances observed to cause seizures and medications that can cause abnormal sodium leading to seizures include all diueretics, selective serotonin reuptake inhibitors (SSRI), tramadol, and serotonin and norepinephrine reuptake inhibitors (SNRI). An electroencephalograph (EEG) is essential for the diagnostic evaluation of epileptic seizures [23,24]. EEG can help predict recurrent seizures. An epileptiform abnormality and focal slowing of background rhythms can be predictive; however, epileptiform discharges have a stronger predictive value [25]. The sensitivity and specificity of interictal epileptiform discharges, in adults, on EEG for recurrent seizures is 17.3% and 94.7%, respectively [26]. In one study, this correlated with a 77% and 47% post-test probability of seizure recurrence with and without noticeable epileptiform discharges on EEG, respectively [25]. It has been supported that epileptiform discharge present on EEG in the setting of a single unprovoked seizure is predictive of recurrent seizure development [25]. Epilepsy is diagnosed after two unprovoked seizures that are not attributable to reversible causes (electrolyte abnormalities, medication or alcohol withdrawals) or traumatic brain injury that occur more than 24 h apart because the risk of recurrence is over 60% [1].

## 5. Partial Onset Seizures in Adults

Pathophysiology, presentation, diagnosis, and risk factors.

Partial seizures manifest as a result of pathogenic activation of a cortical area due to pathological disinhibition. As aforementioned, a complex set of structural and functional changes in neurons, glia, extracellular matrix, and neuro-glia vascular interface can occur and be responsible for partial seizures [27,28]. Similar to epilepsy, these changes caused by a primary insult (i.e., trauma, inflammation, anoxia, genetics, cerebrovascular accident) can result in partial seizure years after the insult. As such, the risk factors for partial seizures are similar to those for epilepsy.

These seizures usually present with neurological findings that can be localized in the affected cortical region. Onset is usually rapid, associated with slowing background activity on EEG, and the seizure only lasts a couple of minutes. They can present with or without loss of consciousness and automatisms. Scalp EEG findings for partial seizures are an initial slowing in background activity followed by a large, irregular amplitude discharge, which transitions into rhythmic bursting followed by electrical depression [29]. Generalized seizures have large, irregular amplitude discharges from beginning to end of the seizure [29]. Diagnosis is made clinically from history, physical, scalp EEG, and the rule out of other possible diagnoses. The criteria for epilepsy diagnosis are aforementioned.

### 5.1. Pharmacologic Treatment

Medical management of epilepsy seeks to eliminate or reduce the frequency of seizures, help patients maintain a normal lifestyle, and maintain psychosocial and occupational activities while avoiding the negative side effects of long-term treatment [30]. The main goal of antiepileptic drug (AED) therapy is to prevent seizures completely with limited toxicity, adverse drug reaction, and consideration of comorbid conditions. In this pursuit, AED therapy should be introduced early, especially for those patients who need to maintain an active lifestyle, with careful consideration of the patient’s type of epilepsy, associated neurological or medical issues, and comorbid conditions [30]. Treatment of new-onset single seizure patients should be carefully considered, and initiation of treatment should be weighed on the probability of recurrent seizures occurring and a diagnosis of epilepsy being made [31].

Carbamazepine, phenytoin, and oxcarbazepine are the current, first-line treatment options for partial seizures. Carbamazepine and phenytoin treat complex partial seizures in adults, and oxcarbazepine can treat partial seizure, simple and complex, in adults [30]. Levetiracetam and tiagabine are suitable for adjunctive treatment options. For every treatment option, careful consideration should be given to side effects and the patient’s comorbidities.

### 5.2. Mechanical Treatment (Vagus Nerve Stimulation)

Vagal nerve stimulation (VNS) was introduced as an adjunctive therapy option in those patients with medication-resistant epilepsy [32]. VNS is a surgical intervention to place a neurocybernetic prosthetic implant that intermittently stimulates the left cervical vagus nerve to suppress seizures [32]. VNS is a lower risk option compared to conventional neurosurgical interventions that attempt to remove a seizure focus region [33]. VNS side effects are coughing and hoarseness, which fluctuate with the level of stimulation provided by the implant. VNS has been around since the 1980s and provides clinicians with a unique, tested therapy that uses the natural peripheral and central connections of the vagus nerve to control treatment-resistant seizures [32].

## 6. Cenobamate

### 6.1. Pharmacological Considerations

Cenobamate, sold under the brand name XCOPRI and also known as CNB, is a newly-FDA approved drug used to treat partial-onset seizures in adult patients. It is distributed as an oral tablet, and dosing starts at 12.5 mg daily. This drug has also been recently approved by the European Commision (EC) and is sold in the European Union as Ontozry. It can be titrated up but is not to exceed 400 mg once daily. Cenobamate acts as a positive allosteric modulator of the GABA_A_ ion channels and is effective in reducing repetitive neuronal firing by inhibition of voltage-gated sodium channels, but the complete mechanism of action is currently unknown. In clinical trials, cenobamate was administered as adjunctive therapy of 100 mg once daily [34]. Cenobamate increased phenytoin mean Cmax and area under the curve (AUC) by 70% and 84%, respectively, and phenobarbital mean Cmax and AUC by 34% and 37%, respectively [34]. Multiple doses of concomitant Cenobamate 200mg once daily decreased carbamazepine mean Cmax and AUC, each by 23% [34]. No clinically significant differences in the pharmacokinetics of the following drugs were seen with concomitant cenobamate treatment: valproic acid, levetiracetam, or lacosamide [34]. The side effects associated with cenobamate use were dose-dependent increases in somnolence, fatigue, dizziness, gait disturbance, coordination disturbance, cognitive dysfunction, confusion, and visual changes [34]. The serious adverse effects associated with cenobamate are drug reaction with eosinophilia and systemic symptoms, QT shortening, suicidal behavior, and suicidal ideation [34]. Initiation of CNB in patient’s taking multiple medication should be carefully considered because some of the most commonly prescribed medications (SSRIs, methadone, macrolides) can cause QT prolongation and arrhythmias can occur. Due to these adverse effects, it is crucial to monitor other drugs or drug interactions that can cause QT interval shortening and CNS depression, including alcohol. Cenobamate must be renally dosed and is not recommended in those with end-stage renal disease [34]. Cenobamate should be used with caution in patients with mild to moderate hepatic impairment and is not recommended in patients with severe hepatic impairment. More research is necessary to further evaluate the use of cenobamate in individuals who are pregnant or lactating and in the pediatric and geriatric populations [34].

### 6.2. Mechanism of Action

Cenobamate increases the inactivation of the fast and slow (transient) voltage-gated sodium channels that facilitate the depolarization period of a neuronal action potential, usually inactivated during repolarization. A percentage of these sodium channels remain active during the downstroke of the action potential and are designated as persistent sodium channels [35]. Cenobamate predominately exerts inhibitory effects on the persistent sodium channels in the CA3 hippocampal neurons, an area with abundant axonal networks, increasing its susceptibility to seizure potential [3]. Persistent sodium channels have the ability to increase the number of recurrent action potentials in the neuron leading to neuronal hyperexcitability [36]. The inhibition of both transient and persistent sodium channels effectively diminishes both avenues of hyperexcitability in an epileptic hippocampus by increasing the rate of entry into the inactivation phase and increasing the refractory period of the action potential [37]. Cenobamate has a greater binding strength to the inactivated state of the sodium channel, causing a more potentiated inactivation state [36]. Cenobamate has the ability to positively potentiate six out of the nineteen recognized GABA_A_ subunits in a concentration-dependent fashion to increase neuronal inhibition in the overexcited pathways of the epileptic hippocampus [37]. Both tonic (outside of the synapse) and phasic (synaptic) GABA_A_ inhibitory neurons are positively potentiated by cenobamate [38,39]. The GABA_A_ subunits which cenobamate binds are distinct from the subunits bound by benzodiazepines and non-benzodiazepines CNS depressants, such as eszopiclone, as demonstrated by the inability of flumazenil to counteract the inhibitory effect of cenobamate [40]. The computation of inhibiting the persistent sodium channels and enhancing GABA_A_ receptor activity in CA3 hippocampal neurons has led to diminished seizure effects [38,41].

### 6.3. Pharmacokinetics

The pharmacokinetic profile of cenobamate has been studied in general and special populations. In one study, single (5 to 75 mg) and multiple (50 to 600 mg/day) oral rising-dose of Cenobamate (capsule formation) in healthy individuals was found to have a maximum plasma concentration between 0.8 and 4 h after oral administration [42]. The area under the plasma concentration-time curve (AUC) after single-dose administration demonstrated that cenobamate increased more than in a dose-proportionate manner; however, after multiple dosing from 50–500 mg/day, the AUC increased in a dose-proportionate manner [42]. There are no significant repercussions in safety and tolerability from the observed plasma accumulation in multiple dosing up to 500 mg/day [42]. For the single dose, cenobamate shows a limited distribution in the body [42]. This is suggested by the oral clearance and elimination rate constants staying analogous over the tested single-dose range. Cenobamate has a half-life of 30 h for a 10 mg single dose, and 76 h for a 750 mg single dose [42]. For both doses, clearance increased in a dose-proportionate manner, and steady-state was attained at approximately two weeks [42]. A second study assessed the pharmacokinetic differences and dosing in renally impaired (RI), hepatic impaired (HI), and elderly (>65 years) populations [37]. All subjects received a single 200 mg oral dose of cenobamate, and the results were compared to young (18–45 years) and healthy adults [37]. The results demonstrated a 1.5-fold increase in AUC for mild to moderate RI individuals, a 2-fold increase in AUC for mild to moderate HI individuals, and no clinically significant findings were found between elderly and young individuals [37]. The differences in clearance for all three groups were insignificant [37]. Conclusively, both doses of cenobamate generally have a mild side effect profile with the occasional treatment-emergent adverse event, regardless of the AUC increasing non-proportionately in the single-dose category [37]. Dosing adjustments need consideration for RI and HI individuals, but not usually for the elderly [37,42].

## 7. Cenobamate Studies and Clinical Trials

### 7.1. Effects of Cenobamate on Voltage-Gated Sodium Channels in Rat Hippocampal CA3 Neurons

This project studied the mode of action of cenobamate in rat hippocampal CA3 pyramidal neurons. Using a whole-cell patch-clamp technique measuring membrane current, the purpose of this study was to evaluate the effect cenobamate had on voltage-gated sodium channels in the acutely isolated hippocampal CA3 neurons, sodium currents by slow voltage-ramps, the voltage dependence of sodium channels, inactivation kinetics of voltage-gated sodium channels, and the excitability of CA3 neurons. Cenobamate had no effect on the time to peak or the weighted decay time constant of transient time to peak. Concerning the effect of cenobamate on voltage dependence when conductance at each voltage was normalized to the maximal conductance, cenobamate concentrations at ≤100 μM did not change the half-maximal voltage for activation (−38.2 ± 4.1 mV and −37.9 ± 4.3 mV in the absence and presence of cenobamate, 0.3 ± 0.2 mV shift, n = 8, *p* = 0.41). The midpoint voltage was shifted for inactivation (V_50,inact_) toward a hyperpolarizing range in a concentration-dependent manner; 100 μM cenobamate shifted the V_50,inact_ from −59.1 ± 3.1 to −65.0 ± 2.6 mV (−5.9 ± 2.6 mV shift, n = 8, *p* < 0.01). Concerning the excitability of CA3 neurons, cenobamate reduced neuronal excitability by inhibiting the non-inactivating persistent component of the current of sodium but had no effect on voltage-gated calcium and potassium channels or excitatory receptors [38].

### 7.2. Effects of Cenobamate on GABA-A Receptor Modulation

A 2019 study analyzed the effect of cenobamate on GABA_A_ receptors and GABA-mediated currents using radioligand binding assays to investigate the binding of the cenobamate on the GABA_A_ receptors sites in rat hippocampal CA3 neurons, dentate gyrus, and mouse and rat CA1 hippocampal neurons. The experiment used whole-cell patch-clamp assays to gather electrophysical recordings. Relative activity of GABA_A_ receptors was analyzed on six human GABA_A_ ion channel subtypes expressed in heterologous cells. Cenobamate did not significantly displace the binding of GABA, muscimol, flunitrazepam, or flumazenil to GABA_A_ receptors. Cenobamate did significantly displace the binding of TBPS radioligand to GABA-gated Cl- channel. Cenobamate in the rat hippocampal neurons significantly increased the GABA-induced current in a concentration-dependent manner. The drug’s potentiation of GABA-induced currents was not affected by flumazenil. In mouse CA1 neurons, cenobamate significantly delayed the decay of evoked inhibitory postsynaptic currents without affecting the peak amplitude. Most prominently in rat CA1 neurons, cenobamate enhanced tonic GABA_A_ currents in a concentration-dependent manner. This effect was also seen to a lesser degree in the rat dentate gyrus [41].

### 7.3. Suppression of the Photoparoxysmal Response in Photosensitive Epilepsy with Cenobamate

One single-blind study evaluated the efficacy of cenobamate by assessing its effect on photoparoxysmal-EEG responses to intermittent photic stimulation (IPS) in adults with photosensitive epilepsy [43]. The participants underwent photic stimulation intermittently under three different eye conditions: eye closure, eyes closed, and eyes opened. This stimulation was done after they were either given a placebo or a dose of cenobamate. Cenobamate was dosed at 100 mg, 250 mg, or 400 mg, with data recorded from a total of six patients. Levels of epileptic activity suppression on EEG were seen in a dose-dependent fashion. Starting with the 100-mg dose level, cenobamate produced partial suppression of IPS sensitivity in 33% of patients under eyes-closed condition. Complete suppression of IPS sensitivity in 25% of patients and partial suppression of IPS sensitivity in 100% when given the 250 mg dose. The 400-mg dose produced complete suppression of IPS sensitivity in 25% of patients, and partial suppression in 50% patients in at least one eye condition [43]. There were no deaths seen in the study, and no treatment-emergent adverse events leading to discontinuation. Adverse effects reported include orthostatic hypotension with syncope in one patient, postural dizziness, and somnolence in three patients. Adverse effects were independent of increased dose levels.

### 7.4. Randomized Phase 2 Study of Adjunctive Cenobamate in Patients with Uncontrolled Focal Seizures

A randomized phase 2 study in 2020 evaluated the efficacy and safety of using 200 mg/d of cenobamate as adjunctive therapy in patients with uncontrolled focal seizures. The course of the study was 12 weeks, which included a 6-week titration phase and a 6-week maintenance phase. Participants were randomized, and the study was designed as double-blind with placebo control. Patients received a 50 mg dose of either cenobamate or placebo once a day. The dose was up titrated by 50 mg per day every two weeks until a target dose of 200 mg was met to start the maintenance phase. Percent change from baseline in focal seizure frequency per 28 days was used as a measurement of the primary efficacy outcome [44]. A total of 201 out of an initial 222 patients completed the study and were included in the intent to treat population. A significant median percent reduction was seen in seizure frequency when comparing the cenobamate-treated and placebo-treated patients. The median focal seizure frequency per 28 days during double-blind treatment decreased from 7.5 at baseline to 3.8 for the cenobamate group and from 5.5 at baseline to 5.0 for the placebo group. This translates to a median percent reduction in seizure frequency per 28 days of 55.6% and 21.5% for cenobamate-treated patients and placebo-treated patients, respectively (*p* < 0.0001) [44]. Adverse events observed in the treatment group throughout this 12-week study include somnolence dizziness, headache, nausea, and fatigue. Somnolence and dizziness were the most frequently reported, both at 22.1%. Urinary tract infections occurred in 8% of the cenobamate treated patients compared to 1.8% of placebo patients, and nasopharyngitis occurred in 6.2% of cenobamate-treated patients compared to 0.9% of placebo patients. Ultimately, cenobamate appeared to cause fairly mild or moderate treatment-emergent adverse events (TEAEs) when compared to the placebo. This is demonstrated through findings showing that TEAEs that occurred in >5% of cenobamate-treated patients with a ≥5% difference over the placebo group included somnolence (22.1% vs. 10.1%), dizziness (20.4% vs. 13.8%), balance disorder (7.1% vs. 0.9%), and nystagmus (9.7% vs. 0%) [44].

### 7.5. Safety and Efficacy of Adjunctive Cenobamate

In addition to the study above, another 2020 study evaluated the efficacy and safety of adjunctive cenobamate in patients with focal seizures. Similarly, the study design was double-blinded, random, and placebo-controlled. A 12-week period divided into a 6-week titration and 6-week maintenance phase was also used. However, in this clinical trial, the groups were assigned dosages 100 mg, 200 mg, 400 mg, or placebo. Efficacy outcomes were assessed by the percent change in 28-day focal seizure frequency and then analyzed with a hierarchal step-down procedure that compared the 400 mg, 200 mg, and 100 mg individually to the placebo [45]. Percent change did not appear to differ significantly between the 200 mg and 400 mg groups, though it did differ from the 100 mg group. This is reflected in the finding that the percentage changes in seizure frequency were −24.0% for the placebo group compared to −35.5% (−62.5 to −15.0%; *p* = 0.0071) for the 100 mg dose group, −55.0% (−73.0 to −23.0%; *p* < 0.0001) for the 200 mg dose group, and −55.0% (−85.0 to −28.0%; *p* < 0.0001) for the 400 mg dose group [45]. Vossler et al., in doing a commentary on this trial, found that the median modal dose reported in the 400 mg group should have actually been 300 mg as opposed to 400 mg. This is attributed to the fact that an antiseizure medication possesses a longer half-life therefore a longer titration period is preferable. On taking this into account, Vossler et al. looked at the results of only the modified, intent to treat treatment phase maintenance phase cohorts and adjusted the dose-response median percent seizure reductions. The outcome of this was reductions in median percent seizures to 25%, 40%, 56%, and 65% for the placebo and CNB 100, 200, and 400 mg cohorts, respectively. The 65% reduction at the maximum dose is greater than that seen in any of the pivotal studies on all the second- and third-generation antiseizure medications [46]. This data is based on an analysis that was reported by Chen et al. in 2018 on the seizure-free rate of first, second, and third-generation antiseizure medications. The adverse events seen in this trial were similar to those seen in others, with TEAEs appearing as relatively mild or moderate (somnolence, dizziness, fatigue, etc.). This is seen in the occurrence of 68% in the placebo group, 55% in the 100 mg group, 66% in the 300 mg group, and 82% in the 400 mg group. Serious events (ataxia, nystagmus, vertigo, etc.) appeared as less of a correlation with dose relation and were seen in 6% of the placebo group, 9% of the 100 mg group, 4% of the 200 mg, and 7% of the 400 mg group [46]. Of note, three severe hypersensitivity reactions occurred during this study. There was one reported case of DRESS in a patient who was assigned the 200 mg/day dose of cenobamate; it is hypothesized that this may be linked to a faster titration protocol than was originally performed [45]. A phase III trial is currently active (ClinicalTrials.gov identifier: NCT02535091) exploring the adverse events, such as DRESS, with the intention of exploring titration relationships. A currently recommended dosing titration schedule is being used, and so far, no cases of DRESS have been reported [35].

### 7.6. Cenobamate: A New Adjunctive

Until this point, the efficacy and safety of the clinical studies discussed were focused on the short-term outcomes. The clinical study discussed in the previous paragraph showed enough positive outlook in the short-term phase to allow for an open-label extension. This allowed for the evaluation of long-term efficacy and safety of cenobamate. In this phase, long term efficacy outcomes were reported as the change in seizure frequency per month, and safety outcomes were reported as adverse events. The target dose of cenobamate was 300 mg/day, with 400 mg set as the maximal limitation. A total of 355 patients from the double-blind study entered the open-label extension. Of these patients, 35.3% discontinued therapy, 15.5% due to lack of efficacy, and 6.8% due to adverse events [47]. Though TEAEs only led to discontinuation in 6.8% of patients, 87.6% of patients experienced some form of TEAE. Serious adverse events were defined as seizures and vertigo; these events were seen in 18.3% of patients. The more common TEAEs occurring in greater than 10% of patients were dizziness, somnolence, headache, diplopia, fatigue, and gait disturbance [47]. In the patients who did not discontinue medication, a median reduction of seizure frequency was reported as 76% at 25–30 months, with 20.2% of patients being completely seizure-free at 25–30 months [47]. There are two phase-3 clinical trials that are still active and exploring long-term efficacy and safety, as well as pharmacokinetic measurements. The first of these was discussed previously in relation to DRESS and is estimated to be completed in December 2020; the other study is estimated to be completed in November 2023 [35]. Table 1 is a summary of the studies discussed in this section.

## 8. Conclusions

Cenobamate inhibits persistent sodium channels and induces GABA_A_ inhibitory currents to control epileptic seizures. The pharmacokinetic profile demonstrates a dose dependent plasma concentration for the multiple dosing category and more than a dose dependent plasma concentration for the single dosing category. Furthermore, a long half-life with limited distribution was observed and dose adjustments are only required for hepatic and renally impaired individuals.

Cenobamate shows promise in that it works on two neuronal ion channels that are commonly associated with epilepsy. Furthermore, it has also proven to be efficacious as an adjunctive therapy in a wide range of epileptic syndromes in either decreasing the seizure frequency or completely suppressing all seizures. In addition to this, the reported adverse effects have appeared to be relatively mild, with the most severe being one reported case of DRESS. This has continued to hold true in terms of long-term outcomes as well. Cenobamate is a relatively new drug that shows potential and merit in the continuing research for an antiepileptic drug that demonstrates superiority and clinical significance.

## Figures and Tables

**Table 1:** Summary of Studies.

Study Type	Author	Groups Studied and Intervention	Results and Findings	Conclusions
Multicenter, single-blind, placebo controlled clinical trial	Kasteleijn-Nolst Trenite et al.	Adults with photosensitive epilepsy underwent intermittent photic therapy after given a single dose of either placebo, 100 mg, 250 mg, or 400 mg cenobamate to assess efficacy, pharmacokinetics, and safety of cenobamate.	Photoparoxysmal-EEG response was reduced with the 250 mg and 400 mg doses of cenobamate compared to the placebo. Plasma concentration values of 201 to 400 measured by area under the plasma concentration-time curve resulted in partial suppression in 4 of 6 (66%) of patients. Furthermore, there were no deaths or serious treatment emergent adverse events that led to discontinuation in this study.	Cenobamate is a possible efficacious therapy in epilepsy, given that it suppressed IPS-PPR response in patients with photosensitive epilepsy. Additionally results demonstrated that cenobamate was well tolerated in doses up to 400 mg.
Multicenter, double blind, placebo-controlled clinical trial	Chung et al.	Two hundred and twenty-two patients with focal seizures and ages from 18–65 were randomized and grouped into a treatment or placebo group. The study lasted 12 weeks and consisted of a 6 week titration and 6 week maintenance phase. Dose of cenobamate was started as 50 mg/d, and eventually titrated up to 200 mg for the maintenance phase.	When compared to the placebo, cenobamate had a greater median percent seizure reduction (55.6% vs. 21.5%, *p* < 0.0001). Of the patients in the treatment group, 28.3% were seizure free in the maintenance phase,. 8.8% in the placebo group were seizure free in the maintenance phase. The most common side effects were somnolence, dizziness, headache, nausea, and fatigue.	When compared to a placebo group, adjunctive therapy with cenobamate significantly improved seizure control in adults with uncontrolled focal seizures and treatment was well tolerated.
Multicenter, double-blind, placebo-controlled, dose-response clinical trial	Krauss et al.	Adults from ages 18–70 with focal seizures were assigned to adjuvant once daily oral cenobamate doses of 100 mg, 200 mg, 400 mg, or placebo. There was a 6-week titration phase to titrate patients to assigned doses and a 6 week maintenance phase.	Median percentage changes in seizure frequency were −24.0% (IQR −45.0 to −7.0%) for the placebo group compared with −35·5% (−62.5 to −15.0%; *p* = 0·0071) for the 100 mg dose group, −55.0% (−73.0 to −23.0%; *p* < 0·0001) for the 200 mg dose group, and −55.0% (−85.0 to −28.0%; *p* < 0.0001) for the 400 mg dose group. Treatment-emergent adverse events occurred more frequently with increasing doses: 70% of patients in the placebo group, 65% in the 100 mg group, 76% in the 200 mg group, and 90% in the 400 mg group. One serious case of DRESS and systemic symptoms occurred in the 200 mg group.	Adjunctive cenobamate is efficacious in reducing focal onset seizures in a dose related fashion. However, treatment-emergent events appear to also increase in a dose-related fasion.
Ongoing open label extension of the YKP3089C013	Chung, French, Krauss, et al.	Patients who completed a double-blind placebo-controlled study enrolled in an open label extension. They were either tapered off the study drug before transitioning to open label treatment or allowed to directly enter without initial tapering. The maximum dose was 400 mg.	Median duration of adjunctive cenobamate exposure was 60.6 months and the median modal daily dose was 200 mg. At the time of analysis, 57.5% remained in the extension. Reasons for discontinuation overall were withdrawal by patient (18.8%), adverse events (9.4%), other (8.7%), and lost to follow-up (2.7%).	In 58% of patients, adjunctive cenobamate was generally well tolerated with long-term treatment.
Whole-cell patch recordings and a patch-clamp amplifier electrophysiology	Nakamura et al.	Rat hippocampal CA3 neurons were isolated to study the effects of cenobamate on voltage gated Na+ channels	Cenobamate inhibited the non-inactivating persistent component of I_Na_ (I_NaP_). In addition, cenobamate binds to voltage-gated Na^+^ channels at the inactivated state with a higher affinity. Cenobamate also accelerated the development of inactivation and stunted recovery from inactivation of voltage-gated Na^+^ channels Cenobamate also increased the threshold for generation of action potentials, and decreased the number of action potentials elicited by depolarizing current injection.	whole-cell patch recordings and a patch-clamp amplifier electrophysiology
Radioligand binding displacement assay	Sharma et al	rat hippocampal CA3 neurons, rat dentate gyrus granule cells (DGGCs), and mouse and rat hippocampal CA1 neurons were studied. Radioligand binding displacement assays were conducted to assess the binding of cenobamate on GABA_A_ receptor sites. Potentiation of GABA-induced currents, as well as effects on both phasic and tonic GABA_A_ currents Were obtained by Electrophysiological recordings	Cenobamate did not significantly displace the binding of GABA, muscimol, flunitrazepam, or Ro-15-1788 (flumazenil) to GABA_A_ receptors. Cenobamate significantly displaced the binding of TBPS radioligand to GABA-gated Cl- channel. In rat hippocampal CA3 neurons, cenobamate (≥30 μM) significantly enhanced GABA-induced current (EC_50_ = 164 μM). the potentiation of GABA-induced currents by cenobamate was not affected by flumazenil. In mouse CA1 neurons, cenobamate significantly delayed the decay of evoked inhibitory postsynaptic currents without altering the peak amplitude. Cenobamate also enhanced tonic GABA_A_ currents in a concentration-dependent manner in rat CA1 neurons and DGGCs	Radioligand binding displacement assay

## Data Availability

Not applicable.
